# Umbilical and Peripheral Venous Catheter-Related Outcomes in Premature Neonates

**DOI:** 10.3390/children12111472

**Published:** 2025-11-01

**Authors:** Nilüfer Okur, Turan Derme, Mehmet Büyüktiryaki, Ufuk Ateş, Suzan Şahin, Şerife Suna Oğuz

**Affiliations:** 1 Division of Neonatology, Gazi Yasargil Training and Education Hospital, Diyarbakir 21000, Turkey; 2 Division of Neonatology, Ankara City Hospital, Ankara 06000, Turkey; turanderme@saglik.gov.tr (T.D.); serifesuna.oguz@saglik.gov.tr (Ş.S.O.); 3 Division of Neonatology, Medipol University Hospital, Istanbul 34000, Turkey; 4 Division of Pediatric Surgery, Ankara University, Ankara 06000, Turkey; uates@ankara.edu.tr; 5 Division of Neonatology, Democracy University Hospital, Izmir 35000, Turkey; suzan.sahin@yok.gov.tr

**Keywords:** UVC, PICC, preterm, catheter, complication, infection

## Abstract

**Highlights:**

**What are the main findings?**

- Umbilical venous catheter (UVC) insertion was faster, required fewer attempts, and was associated with fewer thrombosis and leakage complications compared with peripherally inserted central catheters (PICCs).
- Infection rates were similar between UVC and PICC, but PICC use carried higher risks of mechanical complications and the need for additional vascular access.

**What are the implications of the main findings?**

- UVC should be considered the preferred first-line venous access in preterm infants during the early postnatal period.
- PICC may be a better option after UVC removal when ongoing long-term vascular access is required.

**Abstract:**

Background: Central venous catheters are essential but associated with complications in premature infants. We compared the short-term outcomes of umbilical venous catheter (UVC) versus peripherally inserted central catheter (PICC) as the initial postnatal primary venous access in preterm infants. Methods: Preterm infants with a birth weight ≤ 1500 g within the first postnatal hours were included. Patients were randomly assigned to the UVC or PICC groups. Catheter insertion time, number of attempts, number of operators, duration of catheter use, reason for removal, and overall duration were recorded. Results: A total of 107 premature infants were included, with 63 receiving UVC and 44 receiving PICC. Nineteen infants who initially had UVC placement on day 1 required PICC placement on day 5. The number of attempts, number of operators, and insertion time were significantly higher in the PICC group compared with the UVC group (*p* < 0.001, *p* = 0.002, and *p* = 0.002, respectively). Catheter removal due to thrombosis or leakage occurred in 14.2% of UVC cases versus 40.1% of PICC cases (*p* = 0.002). Conclusion: UVC placement appears to be superior to PICC as the first venous access in preterm infants during the early postnatal period. PICC placement may be less advantageous in the first days of life due to a smaller catheter diameter, technical difficulty, and longer insertion time.

## 1. Introduction

In very-low-birth-weight (VLBW) infants admitted to the neonatal intensive care unit (NICU), central venous catheters (CVCs) are frequently used to maintain vascular access, administer intravenous therapies, and deliver total parenteral nutrition (TPN) [1]. The most commonly used CVCs in NICUs are peripherally inserted central catheters (PICCs) and umbilical venous catheters (UVCs) [2]. Previous studies have demonstrated that VLBW infants with central catheterization achieve greater weight gain, shorter hospital stays, and lower infection rates compared with those managed with repeated peripheral venous access [3].

Immediately after birth, the preferred initial central line is often a UVC, sometimes accompanied by an umbilical arterial catheter (UAC). To date, no high-quality study has provided a definitive answer regarding the optimal duration of UVC placement [4]. While some reports suggest that UVCs may remain in place for up to 14 days [5], clinical practice guidelines recommend considering replacement with another central line if continuous access is required beyond 5–7 days [6]. Shalabi et al. [7] reported that UVC use in preterm infants was associated with an increased risk of late-onset sepsis after a median of five days, necessitating alternative vascular access after removal. In a retrospective cohort study, Butler-O’Hara et al. found that infants in the ≤7-day UVC group experienced 1.0 central line-associated bloodstream infection (CLABSI) per 1000 catheter days, whereas those in the >7-day UVC group had 4.0 CLABSI per 1000 catheter days (*p* < 0.001) [8].

In recent years, PICCs have been increasingly adopted in NICUs. These catheters are typically inserted into a peripheral vein and advanced to the inferior (IVC) or superior vena cava (SVC) at the bedside by trained physicians or nurses [9,10]. Inserted using the Seldinger technique, PICCs can usually remain in place for 2–4 weeks [11].

Nevertheless, both UVCs and PICCs may be complicated by serious adverse events, including thrombosis, thromboembolism, arrhythmias due to malpositioned tips, migration into peritoneal, pleural, or pericardial spaces resulting in ascites, pleural effusion, or cardiac tamponade, as well as portal vein thrombosis, hepatic necrosis, and long-term hepatic dysfunction [12].

The aim of the present study was to compare the most commonly used central venous catheters in VLBW infants in terms of infection rates and usability, and to determine the optimal central venous access route for preterm infants during the early postnatal period. Our hypothesis was that inserting a PICC line instead of a UVC within the first day of life may reduce complications and allow for longer catheter use.

## 2. Materials and Methods

### 2.1. Study Design and Participants

This prospective, randomized controlled trial was conducted in a tertiary NICU over a one-year period, between 13 May 2017 and 1 June 2018. The study was approved by the Local Ethics Committee, and written informed consent was obtained from the parents of all enrolled infants. Informed consent was obtained from the patients’ legal guardians immediately after admission to the intensive care unit, simultaneously with other routine ICU consent forms.

Eligible participants were preterm infants with a birth weight ≤ 1500 g admitted within the first postnatal day. Infants with major congenital anomalies such as osteogenesis imperfecta, hypophosphatasia, severe limb anomalies, or critical congenital heart disease requiring urgent vascular access (e.g., prostaglandin infusion), as well as infants of mothers followed by obstetric specialists due to maternal conditions such as premature rupture of membranes and chorioamnionitis, were excluded from the study to avoid bias in the diagnosis of CLABSI. The inclusion and exclusion criteria of the patients are presented in Table 1.

Primary outcome: Catheter removal due to complications such as catheter-related thrombosis, extravasation, or bleeding.

Secondary outcomes were defined as catheter duration, the number of attempts required for catheter insertion, catheter insertion time, the need for additional vascular access, and infection.

After admission, the study team was notified and patients were randomized using a sealed-envelope method to receive either an umbilical venous catheter (UVC; 05F-L, 38 cm, VYGON, Ecouen, France) or a peripherally inserted central catheter (PICC; 28G/1F, 0.17 × 0.35 mm, VYGON GmbH & Co.K, Aachen, Germany) as the first venous access.

After randomization, in both groups, some catheters could not be used within the first 48 h due to mechanical complications. Since further complications such as infection or thrombosis related to these catheters could not be analyzed, a deviation from the intention-to-treat principle occurred. However, the complications within the first 48 h were analyzed between the two groups.

### 2.2. Catheter Insertion and Follow-Up

**Figure 1:**
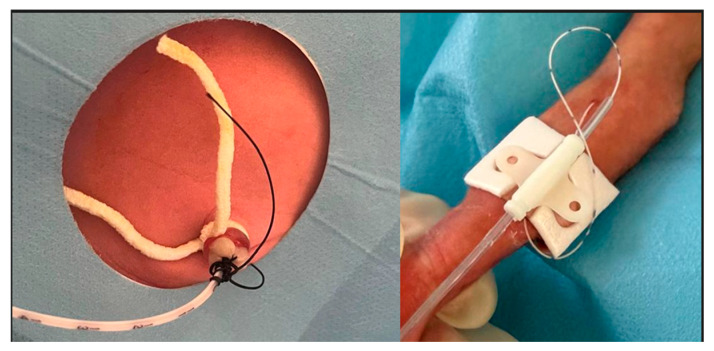
Placement and fixation of Umbilical Venous Catheter (UVC) and Peripherally Inserted Central Catheter (PICC).

According to institutional guidelines [13], UVCs are routinely inserted immediately after birth, whereas PICCs are typically placed during follow-up if vascular access is required. In this trial, infants were randomized to receive either UVC or PICC within the first postnatal hours (Figure 1). All catheters were inserted by an experienced neonatal team. Catheter insertion time was measured with a stopwatch. Catheter position was confirmed by direct radiography and ultrasonography. The UVC was inserted according to the formula: 1.5 × Birth Weight (kg) + 5 cm. The optimal position of the UVC was considered to be between the T8–T9 vertebral levels on direct radiography. Catheters located below T10, or positioned in the hepatic or portal veins, were classified as malpositioned due to the increased risk of extravasation. The tip of the PICC was placed targeting the SVC and IVC. Catheters located in peripheral veins were considered malpositioned. Thrombosis was assessed using Doppler ultrasonography and echocardiography.

### 2.3. Data Collection

Demographic characteristics, including gestational age, birth weight, and sex, were recorded. Procedural variables included catheter insertion time, number of insertion attempts, and number of operators involved. Reasons for catheter removal were classified as infection or feeding intolerance, thrombosis/leakage/occlusion, accidental removal/care error, or no longer required. Feeding intolerance was defined as abdominal distension, vomiting, bilious gastric residuals, and occult or overt bloody stools and was considered a sign of sepsis or necrotizing enterocolitis.

The number of additional catheters required after UVC removal was recorded. Additional vascular access procedures performed through the central catheter for supplementary infusion and blood sampling were recorded and designated as “Number of other invasive attempts.” Catheter-associated bloodstream infections (CLABSI) and causative microorganisms were documented.

CLABSI was defined as the isolation of a pathogen from a blood culture in an infant with a central line in place for ≥48 h. For organisms not commonly found on the skin, a single positive blood culture was sufficient; for organisms commonly found on the skin, at least two separate positive blood cultures were required [10]. When clinical signs of sepsis were observed, blood cultures were obtained prior to initiation of antibiotic therapy. Blood samples were collected by peripheral venipuncture on separate occasions, either on the same or consecutive days, or from different venipuncture sites after appropriate skin preparation. No blood samples were drawn directly from central catheters. Patients were followed until catheter removal.

### 2.4. Statistical Analysis

Baseline characteristics of the study population were summarized using descriptive statistics. Comparisons between UVC and PICC groups were performed using the χ^2^ test for categorical variables and Student’s *t*-test or Mann–Whitney U test for continuous variables, as appropriate. The associations between catheter type, neonatal outcomes, and NICU resource use were also analyzed using the χ^2^ test for categorical variables and the Mann–Whitney U test for continuous variables. The sample size was calculated a priori based on the expected difference in catheter-related complications (thrombosis, leakage, or extravasation) between the UVC and PICC groups. Assuming a two-sided α error of 0.05 and a power of 80% (β = 0.20), it was determined that at least 40 infants per group would be required to detect an absolute difference of 20% in complication rates.

## 3. Results

**Figure 2:**
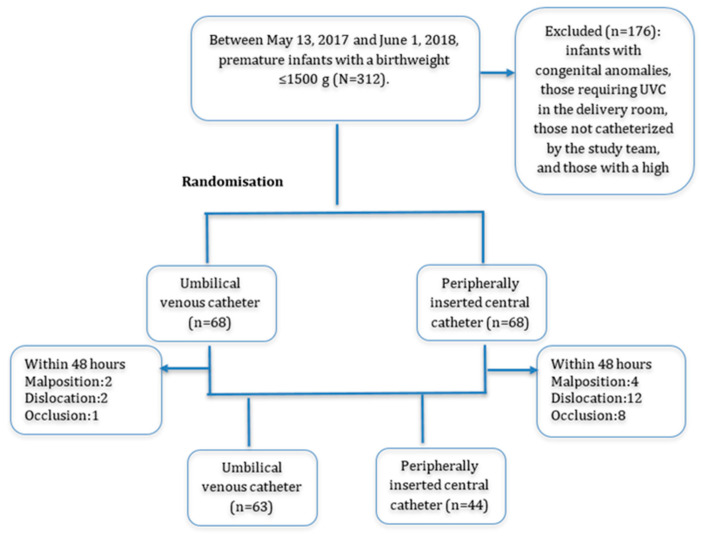
Randomization Flowchart of UVC and PICC Groups.

A total of 312 infants with a birth weight of ≤1500 g were screened during the study period. Of these, 136 infants who met the inclusion criteria were randomized equally into two groups. The UVC group included 68 infants, and the PICC group included 68 infants. Within the first 48 h following catheter insertion, 5 infants in the UVC group and 24 infants in the PICC group required catheter removal due to malposition, occlusion, or dislodgement. The study flow diagram is presented in Figure 2.

Baseline and procedural characteristics of the two groups are summarized in Table 2. Gestational age and birth weight were comparable between the PICC and UVC groups (29.4 ± 1.8 vs. 28.7 ± 2.07 weeks, *p* = 0.083; 1193 ± 146 vs. 1143 ± 188 g, *p* = 0.143). The mean duration of catheter use did not differ significantly between groups (7.5 ± 4.9 vs. 8.2 ± 2.6 days, *p* = 0.319). However, UVC insertion was associated with a significantly shorter procedure time (66 ± 6 vs. 121 ± 15 s, *p* = 0.006) and fewer insertion attempts (median 1 [1–2] vs. 1 [1–4], *p* = 0.003). In addition, the number of other invasive attempts was significantly lower in the UVC group compared with the PICC group (median 0 [0–6] vs. 3 [0–6], *p* < 0.001).

Within the first 48 h after catheter insertion, the catheter became unusable in 5 patients in the UVC group (malposition: 2, dislocation: 2, occlusion: 1) and in 24 patients in the PICC group (malposition: 4, dislocation: 12, occlusion: 8) (*p* < 0.01). These patients required the use of another central catheter type or a peripheral intravenous route. In the subsequent period, 63 patients remained in the UVC group and 44 patients remained in the PICC group for the analysis of other complications. All patients underwent thrombosis screening during catheter follow-up using echocardiography and Doppler ultrasonography. Infective endocarditis was detected in one patient in the PICC group, while portal vein thrombosis was identified in one patient in the UVC group. In addition, a hepatic hematoma was observed in one patient in the UVC group. During the follow-up period, extravasation was significantly higher in the PICC group (11.4%) compared to the UVC group (1.6%) (*p* = 0.048). All other complication rates, including feeding intolerance/clinical sepsis, thrombosis, buckling, maintenance errors, catheter removal due to lack of need, and blood culture positivity, did not differ significantly between the groups. The total rate of catheter removal due to thrombosis, extravasation, or leakage was significantly higher in the PICC group compared with the UVC group (*p* = 0.002). Rates of accidental removal/maintenance error and catheters no longer required were similar between groups (*p* = 0.200 and *p* = 0.055, respectively) (Table 3). After UVC removal, a PICC was inserted in 17 infants (27%) due to the continued need for central venous access.

In the UVC group, 16 catheters (25.3%) were removed due to clinical and/or laboratory signs of infection, compared with 10 catheters (22.7%) in the PICC group (Table 2). The catheter-associated bloodstream infection (CLABSI) rate was calculated as 11.6 per 1000 catheter days in the PICC group and 16.4 per 1000 catheter days in the UVC group.

Empirical antibiotic therapy was initiated according to the most frequently identified microorganisms in our clinic, and in cases of growth, antibiotic treatment was adjusted specifically for the isolated pathogen. When catheter-related thrombosis was detected, the catheter was removed. According to current guidelines, if the thrombus was small, a ‘watch-and-wait’ policy was adopted and the patient was monitored for 5 days. Re-evaluation with echocardiography and Doppler ultrasonography was performed, and if the thrombus persisted, low-molecular-weight heparin therapy was initiated [14]. The majority of thrombosis cases did not require treatment. Only one infant from each group required heparin therapy.

Microorganisms isolated from blood cultures in the UVC group included *Klebsiella pneumoniae* (n = 2), *Staphylococcus epidermidis* (n = 2), *Staphylococcus capitis* (n = 1), *Staphylococcus warneri* (n = 1), and *Enterococcus faecalis* (n = 1). In the PICC group, isolates included *Staphylococcus epidermidis* (n = 3) and *Staphylococcus aureus* (n = 1).

Mortality occurred in four patients in the UVC group and three patients in the PICC group; none of these deaths were directly related to acute catheter complications. One excluded patient died on the first postnatal day, approximately 10 h after PICC insertion, from causes suspected to be catheter-related. However, as autopsy consent was not obtained from the family, a definitive diagnosis could not be established.

## 4. Discussion

In this study, we aimed to evaluate whether umbilical venous catheters (UVCs) or peripherally inserted central catheters (PICCs) are more advantageous as the first intravenous access in preterm infants, and to determine which option should be preferred during the early postnatal follow-up period. Our findings demonstrated no significant difference between the two catheter types regarding catheter-related bloodstream infections (CRBSIs). However, PICCs were technically more difficult to insert and were associated with higher risks of extravasation, occlusion, malposition, and dislocation, leading to an increased need for additional vascular access. When UVCs were used as the initial intravenous route, subsequent transition to a PICC provided longer catheter durability when ongoing vascular access was required. Although central venous catheters are essential in NICU management of premature infants, both mechanical and non-mechanical complications are common [4]. Among non-mechanical complications, CRBSI is one of the most important and is also the most frequent cause of catheter removal [15]. Previous studies have shown that infection risk increases if UVCs are used beyond 5–7 days or PICCs beyond 35 days [8,16,17]. According to the 2022 Infection Control Committee guidelines, the risk of infection rises when UVC use exceeds 5–7 days, and therefore switching to another central line, such as a PICC, is recommended [18].

Konstantinidi et al. studied 71 infants and found no significant difference in nosocomial infections between UVC and PICC groups, although higher colonization rates were detected after catheter removal in the UVC group [2]. Gupta et al. [2] reported that catheters left in place for 7 days or longer were associated with higher complication rates. In our study, the mean dwell time for UVCs was 8.2 ± 2.6 days. There were no catheters left in situ for excessively long durations, and colonization was not assessed, which may explain why we found no difference in infection rates between the two groups. Similarly, Shalabi et al. compared initial UVC and PICC placement in infants born < 30 weeks and reported no difference in CRBSI rates, consistent with our results [7]. Arnst et al. also found no difference between UVCs and PICCs, with an incidence of 8 CLABSIs per 1000 catheter days based on CDC criteria [11]. Reported incidences in the literature vary widely depending on definitions used, ranging from 2.1 to 17 per 1000 catheter days, or 6.0% to 36.8% [19,20]. In our study, infection rates were consistent with published data: 16.4/1000 catheter days in the UVC group and 11.6/1000 catheter days in the PICC group. Coagulase-negative staphylococci remain the most common pathogens associated with central line infections in neonates and the leading cause of late-onset sepsis in VLBW infants [1,11]. In our cohort, coagulase-negative staphylococci and Klebsiella species predominated in the UVC group, while coagulase-negative staphylococci were the most frequent in the PICC group, consistent with prior reports [1,11].

Both catheter types were associated with multiple and sometimes severe complications, including infection, local edema, thrombosis, occlusion, malposition, dislocation, liver abscess, pericardial effusion/tamponade, portal venous thrombosis, pleural effusion, embolization, and non-elective removal [21,22]. Mechanical complications occurred in up to 30% of PICC placements [23], ranging from infiltration, occlusion, thrombosis, and migration to extravasation, phlebitis, and catheter fracture [23]. In a retrospective study of 195 VLBW infants, Salonen et al. compared UVCs and PICCs and found that 40% of catheters were removed non-electively, most often due to suspected infection (n = 42) or dislocation (n = 30). Infants with complications had lower birth weights and gestational ages. Removal rates and reasons were similar between the UVC and PICC groups. Thirty-one infants had confirmed catheter infections, with infection rates per 1000 catheter days higher in UVCs than in PICCs. Multivariable analysis identified gestational age, but not catheter type, as a predictor of infection risk. In our study, however, PICCs were more frequently lost due to mechanical rather than infectious complications. Dongara et al. reported comparable success and complication rates between UVC and PICC insertion, supporting PICCs as a safe alternative to UVCs [20]. Yet, our findings showed that PICCs, being technically smaller and more delicate, were harder to insert, required more time and expertise, and were associated with higher complication rates. Notably, a significant proportion of PICCs became unusable within the first 48 h after placement. Additionally, UVCs were more practical for obtaining blood samples. Overall, our data support that PICCs, although viable, were less favorable than UVCs due to higher rates of early mechanical failure.

Other important complications included pleural and pericardial effusion. Pericardial tamponade is particularly critical, with a mortality rate as high as 75% if untreated. The proximity of catheters to the myocardium and the fragility of neonatal myocardium contribute to this high risk [4,24,25]. In our study, we observed one mortality likely attributable to an untreated tamponade related to a PICC. Thrombosis risk is present with both catheter types [2], and most studies have found no significant difference in thrombotic events between them [2,15]. Prolonged UVC use, however, has been linked to increased risk of thrombosis, particularly portal venous thrombosis (PVT), which can lead to chronic prehepatic portal hypertension (PHT) [26,27,28]. Reported PVT rates vary widely from 4% to 43%. Long-term outcomes of PVT include hypersplenism and thrombocytopenia, with PHT rates up to 40–80% [27,29]. In our study, although thrombosis rates were similar between groups, one infant with a UVC required heparin treatment, and others were followed for portal vein thrombosis.

The strengths of our study include its prospective randomized design, catheter insertion and monitoring by an experienced team, and detailed data collection on insertion time, number of attempts, and reasons for removal. The main limitations were its single-center design, relatively small sample size, and technical limitations in routine Doppler ultrasound and echocardiographic screening for thrombosis, which may have led to underestimation of thrombotic events. In our study, some patients whose catheters were removed within the first 48 h due to mechanical complications were excluded from further analysis because of concerns that their inclusion might alter the rates of complications such as infection. This led to a deviation from the intention-to-treat principle.

Additionally, the use of very fine-lumen PICCs (0.17 × 0.35 mm) may have contributed to higher occlusion rates. The study was conducted nearly 8 years ago, and neonatal guidelines have since been updated. Finally, long-term outcomes such as portal hypertension were not assessed.

## 5. Conclusions

This study demonstrated that the use of PICCs as the first central line in preterm infants was associated with higher rates of mechanical complications and did not reduce the risk of catheter-related infections compared to UVCs. Based on our findings and the available literature, UVCs should not be left in place for longer than 7 days, and if central access is still required thereafter, transition to a PICC is recommended. Moreover, the potential long-term consequences of UVC-related thrombosis, such as portal hypertension, should be considered during follow-up.

## Figures and Tables

**Table 1:** Patients’ inclusion and exclusion criteria for participation in the study.

Inclusion Criteria	Exclusion Criteria
Preterm infants with birth weight ≤ 1500 g	Infants with major congenital anomalies (critical congenital heart disease, osteogenesis imperfecta, hypophosphatasia, severe limb anomalies)
Admitted to the neonatal intensive care unit within the first postnatal day	Infants with probable early neonatal sepsis (premature rupture of membranes and chorioamnionitis)
Postnatal 0–1 h at initiation of procedure	Insertion of an umbilical venous catheter in the delivery room
Postnatal 0–2 h at vascular access	Catheters not inserted by the catheter placement team
Written informed consent obtained from parents	Infants who died (exitus) within the first postnatal day

**Table 2:** Baseline and procedural characteristics of infants in the PICC and UVC groups.

	PICC Group (N = 44)	UVC Group (N = 63)	*p*
Gestational age, week *	29.4 ± 1.8	28.7 ± 2.07	0.083
Birth weight, g *	1193 ± 146	1143 ± 188	0.143
Male, n (%)	22 (50)	32 (50.8)	1.0
Duration of catheter, day *	7.5 ± 4.9	8.2 ± 2.6	0.319
Duration of catheter insertion, s *	121 ± 15	66 ± 6	0.006
Number of attempts **	1 (1–4)	1 (1–2)	0.003
Number of other invasive attempts **	3 (0–6)	0 (0–6)	<0.001

* mean ± standard deviation. ** median (min–max).

**Table 3:** Comparison of safety outcomes observed during follow-up between UVC and PICC groups.

	UVC Group (N = 63)	PICC Group (N = 44)	*p*
Feeding intolerance/clinical sepsis, n (%)	16 (25.3)	10 (22.7)	0.46
Thrombosis, n (%)	7 (11.1)	9 (20.5)	0.17
Extravasation, n (%)	1 (1.6)	5 (11.4)	0.048
Buckling, n (%)	1 (1.6)	4 (9.1)	0.101
Maintenance Error, n (%)	7 (11.1)	2 (4.5)	0.2
No need, n (%)	31 (49.2)	14 (31.3)	0.55
Blood culture positivity, n (%)	7 (11.1)	4 (9)	0.5

## Data Availability

The original contributions presented in the study are included in the article, further inquiries can be directed to the corresponding author.
